# Associations between short-term exposure to gaseous pollutants and pulmonary heart disease-related mortality among elderly people in Chengdu, China

**DOI:** 10.1186/s12940-019-0500-8

**Published:** 2019-07-15

**Authors:** Jianyu Chen, Jie Zeng, Chunli Shi, Ruicong Liu, Rong Lu, Suling Mao, Li Zhang

**Affiliations:** 1 Sichuan Provincial Center for Disease Control and Prevention, No.6, Zhongxue Road, Wuhou District, Chengdu, 610041 China; 2 Chengdu Center for Disease Control and Prevention, Chengdu, China

**Keywords:** Air pollution, Basin, Pulmonary heart disease-related mortality, Elderly people, Time-stratified case-crossover

## Abstract

**Background:**

Pulmonary heart disease (PHD) has become a global burden, especially in low- and middle-income countries. However, very few studies have assessed the influence of air pollution on PHD. This is the first study to explore the association between gaseous pollutants and PHD-related mortality in the central Sichuan Basin of southwestern China.

**Methods:**

Data on PHD-related mortality among elderly people (aged 60 and older) from 2013 to 2017 were collected from the Population Death Information Registration and Management System (PDIRMS). Data on air pollutants were collected from all 24 Municipal Environmental Monitoring Sites in Chengdu, and data on daily temperature, relative humidity, and atmospheric pressure were collected from the Chengdu Municipal Meteorological Bureau. An epidemiological design of time-stratified case-crossover was conducted to assess the association between short-term exposure to ambient gaseous pollutants and PHD-related mortality among elderly people.

**Results:**

About 54,920 PHD-related deaths among people aged 60 and older were reported. After controlling for daily temperature, relative humidity, and atmospheric pressure, an IQR concentration increase in levels of sulfur dioxide (SO_2_) (13 μg/m^3^), nitrogen dioxide (NO_2_) (17 μg/m^3^), and ozone (O_3_) (74 μg/m^3^) was associated with 7.8, 6.2, and 5.5% increases in PHD-related mortality in people aged 60 and older, respectively. People over age 70 might have even higher susceptibility to PHD-related mortality associated with SO_2_, NO_2_, and O_3_. Females and individuals with alternative marital statuses (widowed, divorced, or never married) had twice and more than twice the PHD-related mortality risk associated with SO_2_ and NO_2_ than males and married individuals, respectively.

**Conclusions:**

Increased concentrations of ambient SO_2_, NO_2_, and O_3_ were significantly and positively associated with PHD-related mortality in Chengdu, China. Sociodemographic factors – including gender, age, and marital status – may modify the acute health effects of gaseous pollutants.

**Electronic supplementary material:**

The online version of this article (10.1186/s12940-019-0500-8) contains supplementary material, which is available to authorized users.

## Background

Pulmonary heart disease (PHD) was once described as the overall condition of the right heart from the time it is first affected by pulmonary disease to the final failure [1]. More recently, this definition has been expanded to account for pulmonary arterial hypertension resulting from diseases affecting the structure and/or the function of the lungs. Pulmonary arterial hypertension results in right ventricular enlargement (hypertrophy and/or dilatation) and over time may lead to right heart failure [2]. PHD has emerged as a major cause of disability and mortality within cardiovascular diseases [3]. In the United States, PHD is estimated to account for 7 to 10% of all heart diseases, and to be associated with between 10 and 30% of all hospital admissions for heart failure [4]. Autopsy studies in patients who died of chronic lung disease found that 40% were accompanied with PHD anatomically [5]. PHD has sharply become a global burden, especially in low- and middle-income countries (LMICs) [6]. In developing countries, 20–25 million people have some form of pulmonary vascular disease, which is a much larger number than in the developed world [7].

In recent years, numerous studies were conducted to explore the association between air pollution and cardiovascular disease [8, 9]; these studies focused on coronary heart disease [10], heart failure [11], myocardial infarction [12], and other conditions. However, very few studies assessed the influence of air pollution on PHD. In 1993, by recruiting a study group of 30 patients in Mexico city, Julio Sandoval, et al. [13] found associations between pulmonary arterial hypertension, cor pulmonale, and long-term exposure to domestic wood smoke. In Turkey, research exploring associations between exposure to biomass smoke and tobacco smoke and frequency of pulmonary hypertension in patients with chronic obstructive pulmonary disease (COPD) was conducted from 2000 to 2010 [14].

In light of these previous findings, our study was conducted to (1) assess the association between short-term exposure to ambient gaseous pollutants (SO_2_, NO_2_, carbon monoxide (CO), and O_3_) and PHD-related mortality in people aged 60 and older and (2) determine the modifying effects of sociodemographic factors (e.g., age, gender, and marital status) on PHD-related mortality due to gaseous pollutants.

We conducted our study in Chengdu city, which is in the central Sichuan Basin, China. The Sichuan Basin, a typical basin in southwest China, exhibits severe air pollution [15]. Chengdu city is densely populated with over 15 million people [16]. Therefore, the city is well suited for such a time series study. To our knowledge, no similar reports regarding to the association between ambient gaseous pollutants and PHD-related mortality have come from studies conducted in this region. Thus, ours may fill a gap in knowledge on this topic. An epidemiological design of time-stratified case-crossover was conducted to assess the association between short-term exposure to ambient gaseous pollutants and PHD-related mortality among elderly people in the central Sichuan Basin of China.

## Methods

### Data collection

Data on mortality were collected from the Population Death Information Registration and Management System (PDIRMS), which covers all 20 districts in Chengdu. The death of a resident was confirmed by a hospital or by doctors at the resident’s home; the data on the death were recorded in the system afterwards. A complete record includes name, ID number, gender, nation, marital status, residential address, birth date, date of death, location of death, primary diagnosis for death, second diagnosis for death, and other information. We omitted information that could be used to identify a subject, such as name, ID number, and residential address. Gender, age, and marital status were retained for further analysis. All death records were obtained from the system from January 1, 2013 to December 31, 2017, and records were extracted for further study only if they fulfilled two criteria:)1) if the subject’s age was 60 or older and (2) if the primary diagnosis of death was PHD. The International Classification of Disease, 10th Revision (ICD–10), was used to diagnose PHD (ICD–10 code I27) and related conditions, which included I27.0 (primary pulmonary hypertension), I27.1 (kyphoscoliotic heart disease), I27.2 (other secondary pulmonary hypertension), I27.8 (other specified pulmonary heart diseases), and I27.9 (pulmonary heart disease, unspecified). Pulmonary embolism (ICD–10 code I26) and other diseases of the pulmonary vessels (ICD–10 code I28) were excluded in our study.

**Fig. 1:**
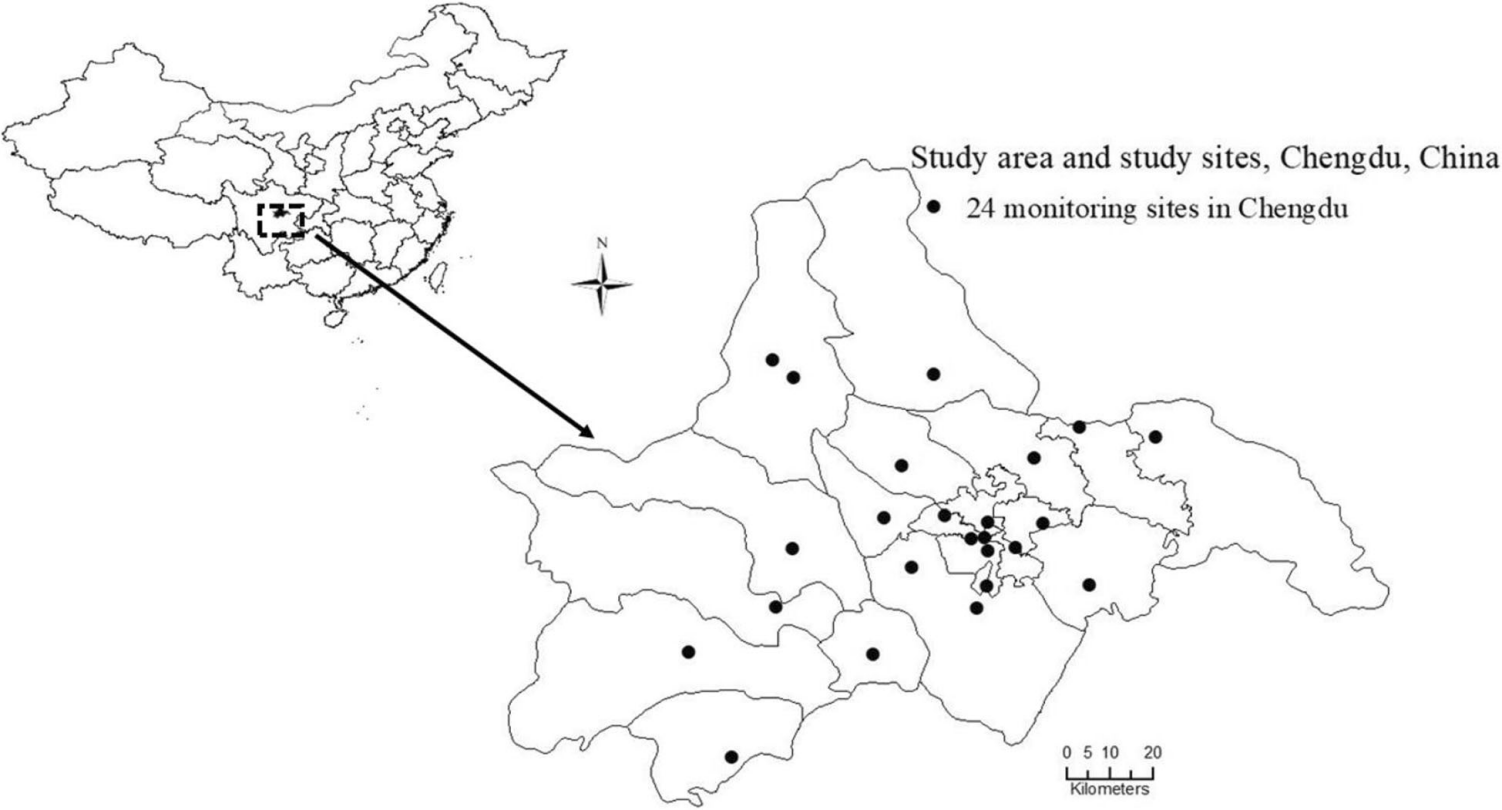
Study area and study locations within the city of Chengdu, China. The enlarged area depicts the spatial distribution of the 24 municipal environmental monitoring sites throughout Chengdu

Data on air pollutants – including SO_2_, NO_2_, CO, daily eight-hour mean concentrations of O_3_ (O3-8h), and particulate matter less than 2.5 μm in aerodynamic diameter (PM_2.5_) – were collected from all 24 Municipal Environmental Monitoring Sites in Chengdu from January 1, 2013 to December 31, 2017 (Fig. 1). Concentrations of air pollutants were continuously monitored 24 h a day, and the data were recorded in the system automatically in every fixed monitoring site. Daily mean concentrations of SO_2_, NO_2_, CO, PM_2.5_, and O_3_-8h were calculated using each pollutant’s data from all 24 sites. Daily 8-h mean concentrations of O_3_ were defined as the highest moving 8-h mean concentrations for O_3_ per day [17]. Missing data of concentrations from one or more sites on a given day were acceptable, and the data from the remaining sites were calculated as mean concentrations for exposure. Weather conditions were considered confounders and needed to be controlled adequately [18]. Data on daily temperature, relative humidity, and atmospheric pressure were collected from the Chengdu Municipal Meteorological Bureau from January 1, 2013 to December 31, 2017.

### Statistical analysis

Spearman’s correlation analysis was used to explore correlations between air pollutants and weather conditions. A method of time-stratified case-crossover was conducted to estimate the association between gaseous pollutants and PHD-related mortality [19, 20]. In using the method of case-crossover, one subject served as both cases and his or her own controls at the same time. In this way, the influences related to individual characteristics – such as age, gender, body mass index, and occupational hazards – could be controlled. The time-stratified method controlled influences such as long-term trends, seasonal patterns, and days of the week by restricting case and controls to the same weekday within the same month and year. The time span between case and controls was limited so that the long-term trends and seasonal patterns would be controlled. Setting control days as the same weekdays mitigated the influence of the day of the week. The number of controls varied between 3 and 4 according to the number of days in the month [21]. For example, if one subject died from PHD on Monday, March 10, 2014, the case day was defined as the same day and the control days were defined as all the remaining Mondays in March 2014 (March 3, 17, 24, and 31), which meant there would be four controls as opposed to only one case.

Delayed effects could exist between exposure to gaseous pollutants and health outcomes [22, 23]. Exposure on a given day could result in death a few days later. To explore the delayed effects, we employed a single-day lag model and the lag days were set from 1 up to 5 prior to the case day.

Weather conditions as confounders – including daily temperature, relative humidity, and atmospheric pressure – were controlled in all models by using natural cubic splines (ns) with three degrees of freedom [24].

The statistical significance of the differences between effect estimates of the strata of a potential effect modifier (e.g. the difference between “male” and “female”) was tested by calculating the 95% confidence interval (95% CI). The equation was (${\hat{Q}}_1-{\hat{Q}}_2$) ± 1.96 $\sqrt{S{\hat{E}}_1^2+S{\hat{E}}_2^2}$, where ${\hat{Q}}_1$ and ${\hat{Q}}_2$ were the estimates for the two categories and $\mathrm{S}{\hat{\mathrm{E}}}_1$ and $\mathrm{S}{\hat{\mathrm{E}}}_2$ were their respective standard errors [25].

Sensitivity analyses were performed in two manners to estimate the robustness of results. First, a two-pollutant model was conducted for pollutants with statistically significant associations with PHD-related mortality. Second, a different lag structure of the multi-day moving average of pollutant concentrations on the case day and up to 3 days prior (Lag01, Lag02, and Lag03, respectively) was conducted for pollutants with statistically significant associations with PHD-related mortality.

The results were calculated as an odds ratio (OR) with a 95% confidence interval (CI) for an interquartile range (IQR) increase in each pollutant. The “Season” package in R (version 3.5.1) was used for fitting the time-stratified case-crossover model [19].

## Results

54,920 PHD-related deaths at age 60 and older were collected from January 1, 2013 to December 31, 2017. 28,971 of the subjects were male and 25,949 were female. 27,424 of the subjects were married and 27,496 were held alternative statuses, including widowed, divorced, and never married. The mean number of daily deaths was 30.1, while the maximum and minimum were 109 and 4, respectively. The mean concentrations of SO_2_, NO_2_, CO, O_3_-8h, and PM_2.5_ were 21.6, 41.5, 1124.2, 92.4, and 70.5 μg/m^3^, respectively, while increases in their IQRs were 13, 17, 498, 74, and 53 μg/m^3^, respectively. The mean daily temperature, relative humidity, and atmospheric pressure were 17.0 °C, 77.7%, and 951.7 hpa, respectively (Table 1).

**Table 1:** Data of air pollutants, weather conditions, and deaths from PHD from 2013 to 2017

	Mean	SD	Min.	25%	50%	75%	Max.	IQR
SO_2_ (μg/m^3^)	21.6	10.5	6.0	14.0	20.0	27.0	71.0	13.0
NO_2_ (μg/m^3^)	41.5	13.2	13.0	32.0	39.0	49.0	89.0	17.0
CO (μg/m^3^)	1124.2	790.2	425.0	787.0	987.0	1285.0	16,608.0	498.0
O_3_-8h (μg/m^3^)	92.4	49.6	11.0	54.0	82.0	128.0	285.0	74.0
PM_2.5_ (μg/m^3^)	70.5	48.9	9.0	36.0	56.0	89.0	372.0	53.0
Temperature (°C)	17.0	7.2	−1.9	10.3	17.9	23.2	30.0	12.9
Humidity (%)	77.7	9.7	41.0	72.0	78.0	85.0	98.0	13.0
AP (hpa)	951.7	7.4	933.1	945.6	951.2	957.4	977.3	11.8
Deaths from PHD	30.1	16.7	4	19	25	37	109	18
Age groups (year)								
60–69	3.3	2.6	0	2	3	4	18	2
70–79	8.8	5.5	0	5	7.5	11	44	6
80–89	13.5	8.0	1	8	11	17	56	9
90–	4.5	3.7	0	2	4	6	25	4
Gender								
Male	15.9	9.6	1	10	13	19	69	9
Female	14.2	8.2	1	9	12	18	57	9
Marital status								
Married	15.0	8.8	1	9	13	18	63	9
Alternative status^a^	15.1	9.1	1	9	13	18	67	9

^a^Alternative marital statuses include widowed, divorced, and never married

Abbreviations: *SO*_*2*_ sulfur dioxide, *NO*_*2*_ nitrogen dioxide, *CO* carbon monoxide, *O*_*3*_*-8h* daily eight-hour mean concentration of O_3_, *PM*_*2.5*_ particulate matter less than 2.5 μm in aerodynamic diameter, *AP* atmospheric pressure, *PHD* pulmonary heart disease, *SD* standard deviation, *IQR* inter-quartile range

Table 2 shows the Spearman’s correlation coefficients of air pollutants and weather conditions. The correlations existed among air pollutants, and between air pollutants and weather conditions. Additionally, correlations of air pollutants existed between each pair of individual sites (Additional file 1: Table S1). Concentrations of SO_2_, NO_2_, CO, and PM_2.5_ were remarkably higher in winter than those in summer, while concentrations of O_3_ were contrary (Additional file 2: Figure S1).

**Table 2:** Spearman’s correlation coefficients of air pollutants and weather conditions

	SO_2_	NO_2_	CO	O_3_	PM_2.5_	Temperature	Humidity	AP
SO_2_	1							
NO_2_	0.474 ^a^	1						
CO	0.615 ^a^	0.678 ^a^	1					
O_3_	−0.047 ^a^	− 0.195 ^a^	− 0.383 ^a^	1				
PM_2.5_	0.622 ^a^	0.785 ^a^	0.817 ^a^	−0.205 ^a^	1			
Temperature	−0.084 ^a^	−0.431 ^a^	− 0.474 ^a^	0.680 ^a^	− 0.462 ^a^	1		
Humidity	−0.446 ^a^	−0.137 ^a^	− 0.058 ^a^	−0.428 ^a^	− 0.190 ^a^	−0.078 ^a^	1	
AP	0.079 ^a^	0.365 ^a^	0.363 ^a^	−0.596 ^a^	0.335 ^a^	−0.851 ^a^	−0.002	1

^a^*P* < 0.05

Abbreviations: *SO*_*2*_ sulfur dioxide, *NO*_*2*_ nitrogen dioxide, *CO* carbon monoxide, *O*_*3*_ ozone, *PM*_*2.5*_ particulate matter less than 2.5 μm in aerodynamic diameter; AP, atmospheric pressure

**Fig. 2:**
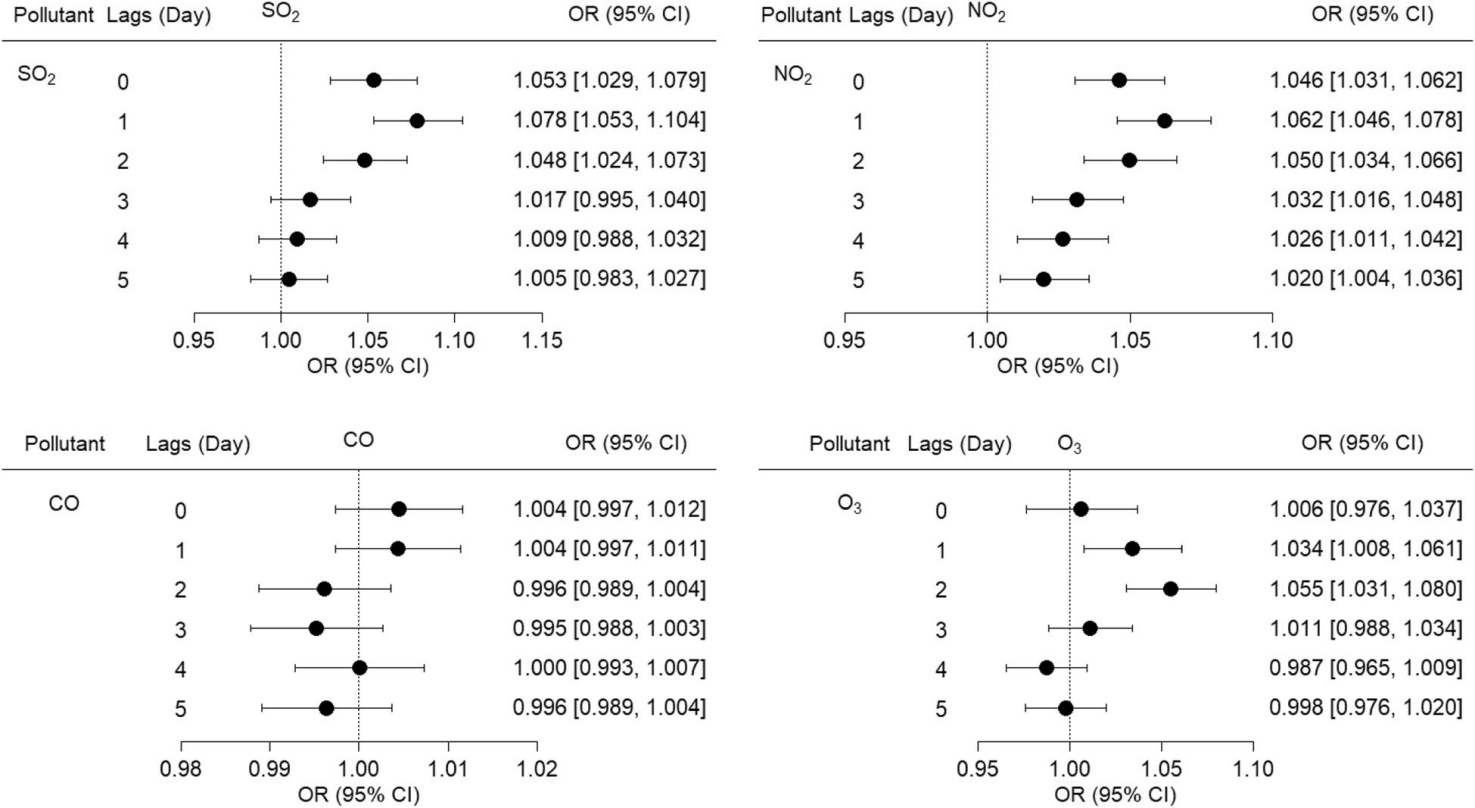
Association between PHD-related mortality among elderly people and IQR increases for SO_2_, NO_2,_ CO, and O_3_ over Lag 0 to Lag 5 days. All models were adjusted for temperature, relative humidity, and atmospheric pressure

Figure 2 shows the calculated results of association between PHD-related mortality in people aged 60 years and older and ambient gaseous pollutants. After controlling the influence of confounders – including daily temperature, relative humidity, and atmospheric pressure – the associations were between PHD-related mortality in people aged 60 years and older and increases in concentration of IQR in SO_2_ (13 μg/m^3^), NO_2_ (17 μg/m^3^), and O_3_ (74 μg/m^3^). The days corresponding to the greatest effects for SO_2_, NO_2,_ and O_3_ were one, one-, and two-day lags, respectively. The association between PHD-related mortality in people aged 60 years and older and CO was not observed. Additionally, an association between PHD-related mortality in people aged 60 years and older and PM_2.5_ was observed in our study. The day corresponding to the greatest effect was one-day lag (Table 3).

**Table 3:** Odds ratios calculated via different lag-day model structures

Air pollutants	Lag (days)	OR	OR 95% CI	
			Lower	upper
SO_2_	0	1.053 ^a^	1.029	1.079
	1	1.078 ^a^	1.053	1.104
	2	1.048 ^a^	1.024	1.073
	3	1.017	0.995	1.040
	01	1.086 ^a^	1.057	1.116
	02	1.095 ^a^	1.064	1.128
	03	1.090 ^a^	1.056	1.124
NO_2_	0	1.046 ^a^	1.031	1.062
	1	1.062 ^a^	1.046	1.078
	2	1.050 ^a^	1.034	1.066
	3	1.032 ^a^	1.016	1.048
	01	1.064 ^a^	1.047	1.082
	02	1.074 ^a^	1.054	1.093
	03	1.077 ^a^	1.056	1.098
CO	0	1.004	0.997	1.012
	1	1.004	0.997	1.011
	2	0.996	0.989	1.004
	3	0.995	0.988	1.003
	01	1.008	0.998	1.017
	02	1.004	0.993	1.015
	03	1.000	0.988	1.013
O_3_	0	1.006	0.976	1.037
	1	1.034 ^a^	1.008	1.061
	2	1.055 ^a^	1.031	1.080
	3	1.011	0.988	1.034
	01	1.037 ^a^	1.000	1.074
	02	1.076 ^a^	1.036	1.117
	03	1.070 ^a^	1.029	1.113
PM_2.5_	0	1.032 ^a^	1.018	1.047
	1	1.038 ^a^	1.023	1.053
	2	1.028 ^a^	1.013	1.042
	3	1.013	0.999	1.027
	01	1.042 ^a^	1.026	1.058
	02	1.045 ^a^	1.027	1.062
	03	1.043 ^a^	1.025	1.062

Abbreviations: *SO*_*2*_ sulfur dioxide, *NO*_*2*_ nitrogen dioxide, *CO* carbon monoxide, *O*_*3*_ ozone, *PM*_*2.5*_ particulate matter less than 2.5 μm in aerodynamic diameter, *OR* odds ratio, *CI* confidence interval

^a^*P* < 0.05

**Fig. 3:**
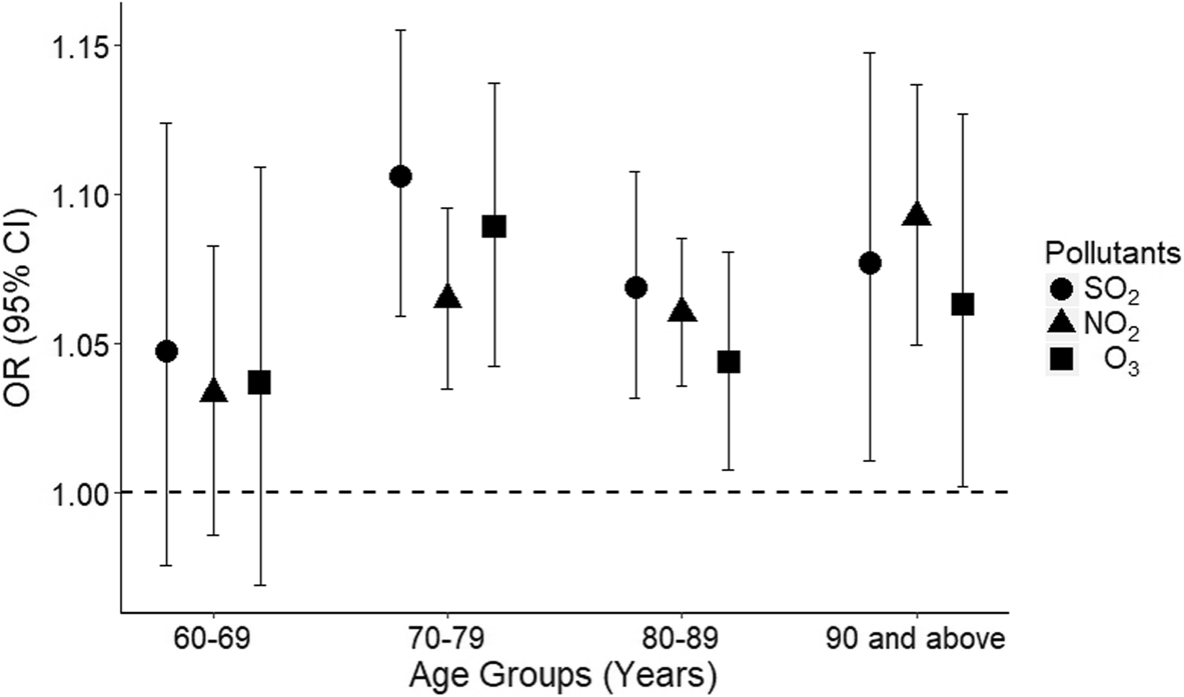
Association between PHD-related mortality among elderly people and IQR increases for SO_2_, NO_2_, and O_3_ among different age groups

Associations between PHD-related mortality in people age 60 years and older and gaseous pollutants varied according to age group. The associations were found in age groups 70 to 79, 80 to 89, and 90 and above, while no association was observed in the age group 60 to 69. The greatest effects for SO_2_, NO_2,_ and O_3_ in age group 70 to 79 were at Lag1 (OR = 1.106, 95% CI: 1.060–1.155), Lag1 (OR = 1.065, 95% CI: 1.035–1.095), and Lag2 (OR = 1.089, 95% CI: 1.043–1.137); in age group 80 to 89 these were at Lag1 (OR = 1.069, 95% CI: 1.032–1.107), Lag2 (OR = 1.060, 95% CI: 1.036–1.085), and Lag2 (OR = 1.043, 95% CI: 1.008–1.081); and in age group 90 and above these were at Lag1 (OR = 1.077, 95% CI: 1.011–1.147), Lag1 (OR = 1.092, 95% CI: 1.049–1.137), and Lag3 (OR = 1.063, 95% CI: 1.002–1.127) (Fig. 3).

**Fig. 4:**
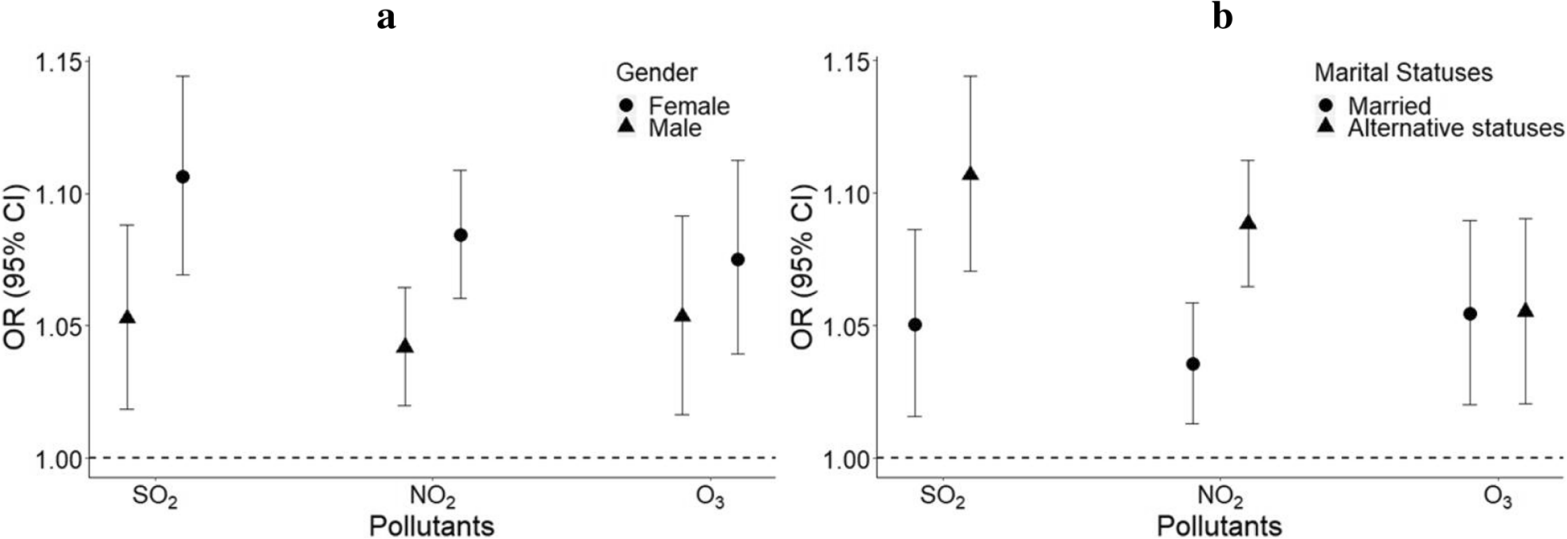
Association between PHD-related mortality among elderly people and IQR increases for SO_2_, NO_2_, and O_3_ between females and males, or between different marital statuses **a**. Between females and males. **b**. Between different marital statuses

For SO_2_, NO_2,_ and O_3_, ORs in female subjects were 1.106 (95% CI: 1.069–1.144), 1.084 (95% CI: 1.060–1.109), and 1.075 (95% CI: 1.039–1.113), respectively; in male subjects these were 1.052 (95% CI: 1.019–1.088), 1.042 (95% CI: 1.020–1.065), and 1.053 (95% CI: 1.017–1.091), respectively; in those with alternative marital statuses (widowed, divorced, or never married) these were 1.107 (95% CI: 1.071–1.144), 1.088 (95% CI: 1.065–1.112), and 1.055 (95% CI: 1.021–1.091), respectively; and in married individuals these were 1.051 (95% CI: 1.016–1.086), 1.036 (95% CI: 1.013–1.059), and 1.055 (95% CI: 1.020–1.090), respectively. In the cases of SO_2_ and NO_2,_ significant statistical differences in the effect estimates existed between females and males and between alternative marital status individuals and married individuals. However, in the case of O_3_, differences between females and males and between alternative marital status individuals and married individuals were not observed (Fig. 4).

For SO_2_, NO_2_, and O_3,_ corresponding to their greatest effective days (SO_2_ at Lag1, NO_2_ at Lag1, and O_3_ at Lag2, respectively), we applied a two-pollutant model by adding PM_2.5_, SO_2_, NO_2_, O_3_, and CO as co-variants, respectively. The health effects of SO_2_, NO_2_ and O_3_ mainly remained in two- pollutant models, although the effects of SO_2_ became null after adjusting for NO_2_ (Table 4).

**Table 4:** Odds ratios (OR) for two-pollutant models including SO_2_, NO_2_, and O_3_^b^

Air pollutants		OR	OR 95% CI	
			Lower	upper
SO_2_		1.079 ^a^	1.053	1.104
	+PM_2.5_	1.062 ^a^	1.031	1.093
	+NO_2_	1.020	0.986	1.055
	+CO	1.078 ^a^	1.053	1.104
	+O_3_	1.075 ^a^	1.050	1.101
NO_2_		1.062 ^a^	1.046	1.078
	+PM_2.5_	1.062 ^a^	1.040	1.084
	+SO_2_	1.052 ^a^	1.029	1.076
	+CO	1.063 ^a^	1.046	1.080
	+O_3_	1.060 ^a^	1.044	1.077
O_3_		1.055 ^a^	1.031	1.080
	+PM_2.5_	1.051 ^a^	1.027	1.076
	+SO_2_	1.047 ^a^	1.023	1.072
	+NO_2_	1.044 ^a^	1.020	1.069
	+CO	1.055 ^a^	1.030	1.080

Abbreviations: *SO*_*2*_ sulfur dioxide, *NO*_*2*_ nitrogen dioxide, *O*_*3*_ ozone, *OR* odds ratio, *CI* confidence interval

^a^*P* < 0.05

^b^ ORs for SO_2_, NO_2_, and O_3_ were from one, one-, and two-day lags, respectively

## Discussion

In using the method of time-stratified case-crossover, we determined a significantly positive association between PHD-related mortality in people aged 60 and older and gaseous pollutants in Chengdu, China. After controlling the influences of confounders – including daily temperature, relative humidity, and atmospheric pressure – an IQR concentrations increase in levels of SO_2_ (13 μg/m^3^), NO_2_ (17 μg/m^3^), and O_3_ (74 μg/m^3^) was associated with 7.8, 6.2, and 5.5% increases in PHD-related mortality in people age 60 and older, respectively.

Compared to previous reported effects of air pollution on cardiovascular mortality, our analysis reported relatively higher effect estimates for the same level of increase in gaseous pollutants concentration. The risks of PHD-relative mortality for SO_2_ were approximately 5.5 times greater than those of cardiovascular mortality derived from the Public Health and Air Pollution in Asia (PAPA) project [26], 2.6 times greater for NO_2_, and 2 times greater for O_3_. Cao et al. [27] reported that the excess risks of cardiovascular mortality per 10 μg/m^3^ increase in SO_2_ and NO_x_ in China were 4.8 and 2.7%, respectively. The risks of PHD-relative mortality in our study were both 1.3 times greater. Compared to the calculated results of risks of cardiovascular mortality derived from the APHEA (Air Pollution and Health: A European Approach) project [28], those of PHD-relative mortality for NO_2_ in our study were 9 times greater. Although the results for risks from the aforementioned studies corresponded to the entire population, and those in our studies corresponded to those of only the elderly population, we assumed that the effects of gaseous pollutants on PHD-related mortality might be higher than those on other cardiovascular mortalities. Further studies are warranted to address these differences.

The major sources of gaseous pollutants comprised of SO_2_, NO_2_, CO, and O_3_ in Chengdu were mainly from industrial emissions and fuel combustion. In addition, NO_2_ and CO can also derive from traffic emissions [29, 30]. The concentrations of gaseous pollutants showed typically seasonal trends in our study. The concentrations of SO_2_, NO_2_, and CO were remarkably higher during winter each year, which may be due to the reduction of air flow diffused dilution efficiency and to the increase of biomass fuel combustion for heating. Higher concentrations of O_3_ in summer were probably caused by increased sunlight. However, the aforementioned seasonal changes were controlled by using the case-crossover method.

The pathogeny of PHD is multifarious [2]. As confirmed, chronic obstructive pulmonary disease (COPD) is the primary disease leading to PHD, accounting for approximately 80% of cases [3]. COPD could lead to hypoxic pulmonary vasoconstriction, polycythemia, and destruction of the pulmonary vascular bed [31], while also causing impaired lung function and/or structural damage [32], resulting in pulmonary hypertension [33]. Furthermore, gaseous pollutants—including SO_2_, NO_2,_ and O_3_—have been associated with COPD in short-term exposure studies, long-term exposure studies, and in outdoor or indoor exposure studies [34–36]. These findings might provide a reasonable explanation for the exacerbation of PHD-related mortality in people with COPD with increasing concentrations of gaseous pollutants. The major cause of death associated with PHD was right heart failure [2]. According to previous studies, heart failure-related hospitalization or death was associated with increases in SO_2_ (2.36% per 10 parts per billion) and NO_2_ (1.70% per 10 parts per billion) [11]. Thus, increased concentrations of gaseous pollutants might exacerbate PHD-related mortality by inducing heart failure in PHD patients, which might also explain how gaseous pollutants exacerbate PHD-related mortality.

Our study found significant statistical evidence for the modification of PHD-related mortality by age, gender, or marital status. As reported in previous studies, elderly people seemed to have higher susceptibility to mortality caused by gaseous pollutants [37, 38], which was in line with our findings. In our study, the association between gaseous pollutants—including SO_2_, NO_2,_ and O_3_—and PHD-related mortality was observed in individuals aged 70 or older but not in those ranging from 60 to 69 years old, indicating that people aged 70 and older might have higher susceptibility to the health effects of gaseous pollutants. Although previous studies found little evidence for a higher occurrence of cardio-respiratory mortality among women than men [39], we determined that females might be more susceptible to PHD-related mortality associated with gaseous pollutants than males. The risk of PHD-related mortality due to exposure to SO_2_ and NO_2_ among females was twice that of males. Marital status also could influence health and mortality [40]. In line with previous studies, we found that individuals with alternative marital statuses (widowed, divorced, or never married) had a higher risk of PHD-related mortality due to gaseous pollutants, including SO_2_ and NO_2_, than married people. The effect estimates among individuals with alternative marital statuses were more than twice those of married individuals.

According to the results from the two-pollutant models, NO_2_ and O_3_ seemed to have more stable health impacts on PHD-related mortality than SO_2_. After adjusting for another pollutant, the effects of PHD-related mortality caused by NO_2_ and O_3_ remained with similar values of ORs calculated from single pollutant models that, when caused by SO_2_, attenuated to be negative after adjusting for NO_2_. Renjie Chen et al. [37] suggested that SO_2_ may serve as a surrogate for other toxic substances correlated with NO_2_, which may explain how NO_2_ reduces the association of SO_2_ with mortality. Thus, NO_2_ and O_3_ might merit more attention in regard to their association with PHD due to ambient gaseous pollutants.

In sensitivity analyses, ORs calculated via different lag-day structures (Lag01, Lag02, and Lag03, respectively) were similar to those from the single-day lag models (Table 3). The results of the models were reliable.

Our study has three main strengths: First, this is the first study to explore the association between gaseous pollutants and PHD-related mortality in the central Sichuan Basin of southwestern China. Second, mortality data for the whole city were used in our study due to the advantage of the PDIRMS; thus, the data were authentic and reliable. Third, the city of Chengdu is large and densely populated, which is advantageous for performing an ecological study that assesses the effects of air pollutants on health. Our study also has some limitations. First, concentrations of air pollutants were obtained from municipal environmental monitoring sites that are fixed; thus, individual exposure data were not evaluated. Second, this study was conducted in central Sichuan Basin; consequently, the basin’s specific geographical features and weather conditions should be considered and generalizations of these results should be treated cautiously. Third, we conducted a time-stratified case-crossover analysis based on individuals from whom cumulative effects could not be obtained.

## Conclusion

Our study found that increased concentrations of ambient SO_2_, NO_2_, and O_3_ were significantly and positively associated with PHD-related mortality in Chengdu, China. Furthermore, our results suggest that sociodemographic factors – including gender, age, and marital status – may modify the acute health effects of gaseous pollutants. Our study builds evidence for potential health risks from ambient gaseous pollutants.

## Additional files

Additional file 1: **Table S1.** Spearman’s correlation coefficients of air pollutants between individual monitoring sites. (DOCX 37 kb)

Additional file 2: **Figure S1.** Seasonal trends of monthly concentrations of SO, NO_2_, CO, O_3_, and PM_2.5_ from 2013 to 2017. (PDF 128 kb)

## Data Availability

The datasets used in this study are available from the corresponding author upon reasonable request.

## Abbreviations

AP: Atmospheric pressure
CI: Confidence interval
CO: Carbon monoxide
IQR: Interquartile range
NO_2_: Nitrogen dioxide
O_3_: Ozone
O_3_-8h: Daily eight-hour mean concentration for O_3_
OR: Odds ratio
PHD: Pulmonary heart disease
PM_2.5_: Particulate matter less than 2.5 μm in aerodynamic diameter
SD: Standard deviation
SO_2_: Sulfur dioxide
