# Supplementary material for: Associations between short-term exposure to gaseous pollutants and pulmonary heart disease-related mortality among elderly people in Chengdu, China
Source: Environ Health. 2019 Jul 15;18:64. doi: 10.1186/s12940-019-0500-8 (PMC6632202; doi:10.1186/s12940-019-0500-8)
Supplement: Supplementary file 1 — Table S1. Spearman’s correlation coefficients of air pollutants between individual monitoring sites. (DOCX 37 kb) [file 12940_2019_500_MOESM1_ESM.docx]

Table S1. Spearman’s correlation coefficients of air pollutants between individual monitoring sites ^a^

| Pollutants | Monitoring sites | Min. | 25% | Median | 75% | Max. | IQR |
| --- | --- | --- | --- | --- | --- | --- | --- |
| SO_2_ | Chenghua | 0.330 | 0.509 | 0.555 | 0.637 | 0.819 | 0.128 |
|  | Chongzhou | 0.397 | 0.487 | 0.525 | 0.559 | 0.689 | 0.072 |
|  | Dayi | 0.266 | 0.382 | 0.447 | 0.491 | 0.544 | 0.109 |
|  | Dujiangyan1 | 0.209 | 0.330 | 0.393 | 0.423 | 0.498 | 0.093 |
|  | Dujiangyan2 | 0.170 | 0.333 | 0.359 | 0.450 | 0.513 | 0.117 |
|  | Gaoxin1 | 0.359 | 0.522 | 0.543 | 0.604 | 0.954 | 0.081 |
|  | Gaoxin2 | 0.293 | 0.471 | 0.519 | 0.676 | 0.866 | 0.205 |
|  | Jinniu | 0.364 | 0.503 | 0.582 | 0.671 | 0.855 | 0.169 |
|  | Jintang | 0.328 | 0.440 | 0.489 | 0.539 | 0.619 | 0.099 |
|  | Jinjiang | 0.299 | 0.387 | 0.479 | 0.569 | 0.757 | 0.182 |
|  | Longquan | 0.206 | 0.496 | 0.550 | 0.611 | 0.770 | 0.115 |
|  | Pengzhou | 0.361 | 0.485 | 0.572 | 0.636 | 0.689 | 0.151 |
|  | Pidu | 0.343 | 0.512 | 0.582 | 0.639 | 0.675 | 0.127 |
|  | Pujiang | 0.180 | 0.338 | 0.372 | 0.467 | 0.527 | 0.129 |
|  | Qingbaijiang | 0.267 | 0.487 | 0.527 | 0.592 | 0.769 | 0.106 |
|  | Qingyang1 | 0.337 | 0.497 | 0.590 | 0.688 | 0.866 | 0.191 |
|  | Qingyang2 | 0.324 | 0.495 | 0.555 | 0.630 | 0.820 | 0.135 |
|  | Qionglai | 0.216 | 0.380 | 0.430 | 0.503 | 0.545 | 0.123 |
|  | Shuangliu1 | 0.372 | 0.532 | 0.622 | 0.651 | 0.709 | 0.119 |
|  | Shuangliu2 | 0.293 | 0.470 | 0.523 | 0.594 | 0.742 | 0.124 |
|  | Wenjiang | 0.357 | 0.473 | 0.518 | 0.565 | 0.672 | 0.092 |
|  | Wuhou | 0.170 | 0.381 | 0.505 | 0.554 | 0.954 | 0.173 |
|  | Xindu | 0.337 | 0.533 | 0.613 | 0.652 | 0.770 | 0.120 |
|  | Xinjin | 0.358 | 0.536 | 0.623 | 0.654 | 0.709 | 0.118 |
| NO_2_ | Chenghua | 0.287 | 0.539 | 0.619 | 0.747 | 0.877 | 0.209 |
|  | Chongzhou | 0.453 | 0.524 | 0.540 | 0.576 | 0.686 | 0.051 |
|  | Dayi | 0.221 | 0.386 | 0.406 | 0.452 | 0.614 | 0.066 |
|  | Dujiangyan1 | 0.291 | 0.372 | 0.419 | 0.449 | 0.576 | 0.077 |
|  | Dujiangyan2 | 0.244 | 0.439 | 0.496 | 0.560 | 0.718 | 0.121 |
|  | Gaoxin1 | 0.315 | 0.450 | 0.524 | 0.651 | 0.760 | 0.201 |
|  | Gaoxin2 | 0.245 | 0.521 | 0.610 | 0.675 | 0.815 | 0.154 |
|  | Jinniu | 0.269 | 0.434 | 0.579 | 0.685 | 0.828 | 0.251 |
|  | Jintang | 0.248 | 0.530 | 0.561 | 0.665 | 0.743 | 0.135 |
|  | Jinjiang | 0.275 | 0.484 | 0.614 | 0.719 | 0.877 | 0.236 |
|  | Longquan | 0.259 | 0.448 | 0.494 | 0.530 | 0.657 | 0.082 |
|  | Pengzhou | 0.286 | 0.476 | 0.568 | 0.609 | 0.688 | 0.133 |
|  | Pidu | 0.316 | 0.488 | 0.577 | 0.629 | 0.701 | 0.142 |
|  | Pujiang | 0.245 | 0.280 | 0.315 | 0.362 | 0.529 | 0.083 |
|  | Qingbaijiang | 0.221 | 0.386 | 0.447 | 0.488 | 0.571 | 0.102 |
|  | Qingyang1 | 0.251 | 0.539 | 0.616 | 0.743 | 0.853 | 0.205 |
|  | Qingyang2 | 0.317 | 0.540 | 0.601 | 0.690 | 0.796 | 0.150 |
|  | Qionglai | 0.372 | 0.483 | 0.535 | 0.584 | 0.718 | 0.101 |
|  | Shuangliu1 | 0.375 | 0.501 | 0.595 | 0.648 | 0.681 | 0.147 |
|  | Shuangliu2 | 0.364 | 0.504 | 0.535 | 0.599 | 0.678 | 0.095 |
|  | Wenjiang | 0.291 | 0.522 | 0.555 | 0.582 | 0.656 | 0.060 |
|  | Wuhou | 0.324 | 0.451 | 0.562 | 0.640 | 0.749 | 0.189 |
|  | Xindu | 0.284 | 0.399 | 0.464 | 0.521 | 0.596 | 0.122 |
|  | Xinjin | 0.387 | 0.527 | 0.586 | 0.612 | 0.647 | 0.086 |
| CO | Chenghua | 0.392 | 0.579 | 0.599 | 0.686 | 0.770 | 0.108 |
|  | Chongzhou | 0.476 | 0.629 | 0.690 | 0.742 | 0.787 | 0.114 |
|  | Dayi | 0.540 | 0.602 | 0.703 | 0.753 | 0.813 | 0.151 |
|  | Dujiangyan1 | 0.296 | 0.422 | 0.456 | 0.498 | 0.613 | 0.076 |
|  | Dujiangyan2 | 0.535 | 0.588 | 0.651 | 0.696 | 0.773 | 0.108 |
|  | Gaoxin1 | 0.452 | 0.519 | 0.598 | 0.634 | 0.716 | 0.115 |
|  | Gaoxin2 | 0.434 | 0.675 | 0.720 | 0.753 | 0.838 | 0.078 |
|  | Jinniu | 0.428 | 0.586 | 0.637 | 0.710 | 0.776 | 0.124 |
|  | Jintang | 0.457 | 0.670 | 0.716 | 0.741 | 0.784 | 0.071 |
|  | Jinjiang | 0.441 | 0.632 | 0.698 | 0.723 | 0.798 | 0.091 |
|  | Longquan | 0.316 | 0.584 | 0.616 | 0.667 | 0.710 | 0.083 |
|  | Pengzhou | 0.474 | 0.618 | 0.671 | 0.722 | 0.791 | 0.104 |
|  | Pidu | 0.456 | 0.634 | 0.666 | 0.719 | 0.785 | 0.085 |
|  | Pujiang | 0.508 | 0.584 | 0.657 | 0.716 | 0.813 | 0.132 |
|  | Qingbaijiang | 0.331 | 0.625 | 0.652 | 0.708 | 0.763 | 0.083 |
|  | Qingyang1 | 0.386 | 0.563 | 0.627 | 0.690 | 0.790 | 0.127 |
|  | Qingyang2 | 0.509 | 0.730 | 0.770 | 0.785 | 0.846 | 0.055 |
|  | Qionglai | 0.432 | 0.600 | 0.644 | 0.694 | 0.741 | 0.094 |
|  | Shuangliu1 | 0.296 | 0.538 | 0.560 | 0.622 | 0.685 | 0.084 |
|  | Shuangliu2 | 0.416 | 0.664 | 0.676 | 0.719 | 0.773 | 0.056 |
|  | Wenjiang | 0.481 | 0.713 | 0.745 | 0.758 | 0.846 | 0.045 |
|  | Wuhou | 0.445 | 0.644 | 0.682 | 0.738 | 0.808 | 0.094 |
|  | Xindu | 0.474 | 0.622 | 0.697 | 0.727 | 0.811 | 0.106 |
|  | Xinjin | 0.548 | 0.644 | 0.697 | 0.742 | 0.776 | 0.099 |
| O_3_ | Chenghua | 0.664 | 0.914 | 0.927 | 0.942 | 0.960 | 0.028 |
|  | Chongzhou | 0.839 | 0.897 | 0.914 | 0.925 | 0.948 | 0.029 |
|  | Dayi | 0.836 | 0.900 | 0.913 | 0.923 | 0.965 | 0.023 |
|  | Dujiangyan1 | 0.739 | 0.796 | 0.814 | 0.819 | 0.852 | 0.023 |
|  | Dujiangyan2 | 0.585 | 0.647 | 0.817 | 0.835 | 0.877 | 0.189 |
|  | Gaoxin1 | 0.585 | 0.900 | 0.916 | 0.935 | 0.963 | 0.035 |
|  | Gaoxin2 | 0.620 | 0.911 | 0.927 | 0.934 | 0.966 | 0.023 |
|  | Jinniu | 0.629 | 0.897 | 0.927 | 0.932 | 0.952 | 0.035 |
|  | Jintang | 0.796 | 0.894 | 0.914 | 0.927 | 0.944 | 0.033 |
|  | Jinjiang | 0.612 | 0.913 | 0.930 | 0.942 | 0.968 | 0.029 |
|  | Longquan | 0.739 | 0.842 | 0.877 | 0.898 | 0.924 | 0.056 |
|  | Pengzhou | 0.819 | 0.858 | 0.878 | 0.889 | 0.933 | 0.031 |
|  | Pidu | 0.819 | 0.893 | 0.910 | 0.924 | 0.946 | 0.031 |
|  | Pujiang | 0.798 | 0.846 | 0.872 | 0.887 | 0.938 | 0.041 |
|  | Qingbaijiang | 0.762 | 0.855 | 0.878 | 0.902 | 0.934 | 0.047 |
|  | Qingyang1 | 0.610 | 0.901 | 0.926 | 0.940 | 0.965 | 0.039 |
|  | Qingyang2 | 0.609 | 0.908 | 0.926 | 0.934 | 0.970 | 0.026 |
|  | Qionglai | 0.828 | 0.883 | 0.896 | 0.913 | 0.965 | 0.030 |
|  | Shuangliu1 | 0.816 | 0.905 | 0.929 | 0.952 | 0.966 | 0.047 |
|  | Shuangliu2 | 0.793 | 0.891 | 0.924 | 0.944 | 0.968 | 0.053 |
|  | Wenjiang | 0.818 | 0.896 | 0.915 | 0.927 | 0.954 | 0.031 |
|  | Wuhou | 0.799 | 0.901 | 0.922 | 0.952 | 0.970 | 0.051 |
|  | Xindu | 0.794 | 0.887 | 0.911 | 0.931 | 0.945 | 0.044 |
|  | Xinjin | 0.814 | 0.901 | 0.928 | 0.936 | 0.952 | 0.035 |
| PM_2.5_ | Chenghua | 0.753 | 0.856 | 0.914 | 0.943 | 0.963 | 0.087 |
|  | Chongzhou | 0.774 | 0.839 | 0.854 | 0.885 | 0.943 | 0.047 |
|  | Dayi | 0.797 | 0.832 | 0.864 | 0.889 | 0.949 | 0.057 |
|  | Dujiangyan1 | 0.707 | 0.727 | 0.739 | 0.763 | 0.821 | 0.036 |
|  | Dujiangyan2 | 0.739 | 0.759 | 0.778 | 0.804 | 0.835 | 0.045 |
|  | Gaoxin1 | 0.728 | 0.833 | 0.881 | 0.931 | 0.950 | 0.098 |
|  | Gaoxin2 | 0.760 | 0.878 | 0.914 | 0.939 | 0.954 | 0.061 |
|  | Jinniu | 0.733 | 0.872 | 0.906 | 0.949 | 0.966 | 0.077 |
|  | Jintang | 0.720 | 0.854 | 0.876 | 0.889 | 0.904 | 0.035 |
|  | Jinjiang | 0.733 | 0.839 | 0.900 | 0.939 | 0.956 | 0.101 |
|  | Longquan | 0.707 | 0.822 | 0.881 | 0.902 | 0.934 | 0.080 |
|  | Pengzhou | 0.739 | 0.825 | 0.841 | 0.867 | 0.943 | 0.042 |
|  | Pidu | 0.785 | 0.861 | 0.885 | 0.901 | 0.965 | 0.040 |
|  | Pujiang | 0.785 | 0.829 | 0.846 | 0.860 | 0.940 | 0.032 |
|  | Qingbaijiang | 0.708 | 0.843 | 0.898 | 0.908 | 0.929 | 0.065 |
|  | Qingyang1 | 0.710 | 0.830 | 0.885 | 0.940 | 0.966 | 0.110 |
|  | Qingyang2 | 0.757 | 0.849 | 0.893 | 0.941 | 0.961 | 0.092 |
|  | Qionglai | 0.809 | 0.834 | 0.855 | 0.876 | 0.949 | 0.043 |
|  | Shuangliu1 | 0.754 | 0.845 | 0.869 | 0.888 | 0.914 | 0.043 |
|  | Shuangliu2 | 0.731 | 0.866 | 0.899 | 0.923 | 0.964 | 0.057 |
|  | Wenjiang | 0.760 | 0.880 | 0.903 | 0.921 | 0.965 | 0.041 |
|  | Wuhou | 0.726 | 0.852 | 0.928 | 0.943 | 0.964 | 0.092 |
|  | Xindu | 0.707 | 0.866 | 0.898 | 0.921 | 0.934 | 0.055 |
|  | Xinjin | 0.739 | 0.853 | 0.877 | 0.894 | 0.938 | 0.041 |

Abbreviations: SO_2_, sulfur dioxide; NO_2_, nitrogen dioxide; CO, carbon monoxide; O_3_, ozone; PM_2.5_, particulate matter less than 2.5 μm in aerodynamic diameter; IQR, inter-quartile range

^a^ All results *P* < 0.05.
