# Supplementary figures and images for: Associations between short-term exposure to gaseous pollutants and pulmonary heart disease-related mortality among elderly people in Chengdu, China
Source: Environ Health. 2019 Jul 15;18:64. doi: 10.1186/s12940-019-0500-8 (PMC6632202; doi:10.1186/s12940-019-0500-8)

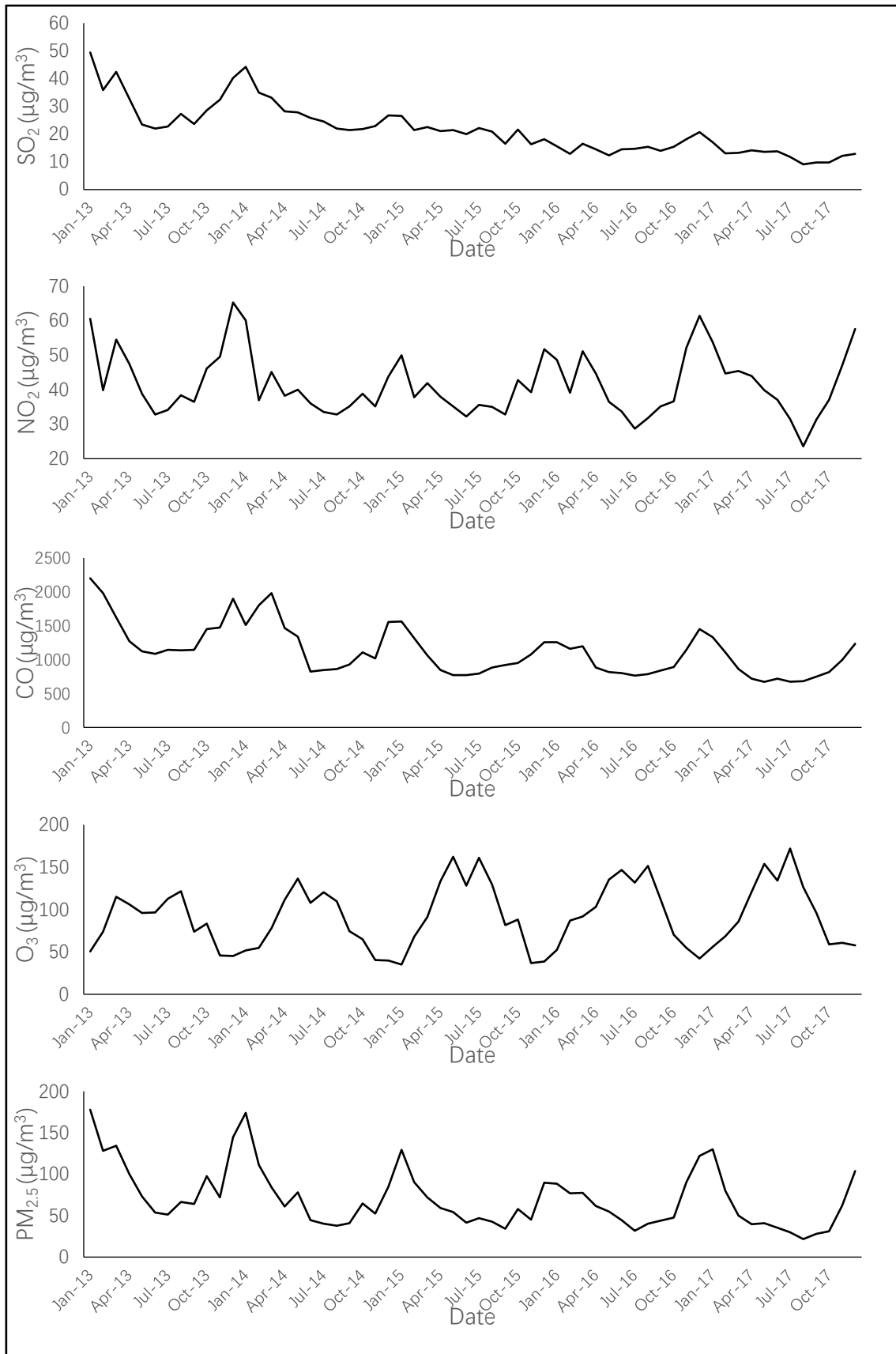

Supplement: Supplementary file 2 — Figure S1. Seasonal trends of monthly concentrations of SO, NO2, CO, O3, and PM2.5 from 2013 to 2017. (PDF 128 kb) [file 12940_2019_500_MOESM2_ESM.pdf]
